# Myocardial Work Index Dispersion as a Potential Predictor of Ventricular Arrhythmias in Patients with Heart Failure with Reduced Ejection Fraction

**DOI:** 10.3390/biomedicines14102159

**Published:** 2026-09-24

**Authors:** Martyna Dąbrowska, Magdalena Potapowicz-Krysztofiak, Patrycja Życzkowska, Małgorzata Maciorowska, Zbigniew Orski, Aleksandra Winkler, Paweł Krzesiński, Marek Kiliszek, Beata Uziębło-Życzkowska

**Affiliations:** 1 Department of Cardiology and Internal Diseases, Military Institute of Medicine–National Research Institute, 04-141 Warsaw, Polandbuzieblo-zyczkowska@wim.mil.pl (B.U.-Ż.); 2 Department of Medicine, School of Medicine and Surgery, University of Padua, 35128 Padua, Italy

**Keywords:** heart failure with reduced ejection fraction, myocardial work, myocardial work index dispersion, ventricular arrhythmia risk

## Abstract

**Background:** Mechanical dispersion of left ventricular contraction reflects electromechanical heterogeneity and may contribute to arrhythmogenesis. We evaluated whether myocardial work dispersion is associated with ventricular arrhythmia (VA) risk in patients with heart failure with reduced ejection fraction (HFrEF). **Methods:** Eighty-five patients with HFrEF and no history of atrial fibrillation of any type and with complete arrhythmia follow-up (mean age 65 ± 12 years, 83.5% male, mean LVEF 30.5 ± 6.6%) underwent echocardiography one day before elective implantable cardioverter-defibrillator (ICD) or cardiac resynchronisation therapy (CRT) implantation and were followed for one year. **Results:** VAs—sustained or non-sustained ventricular tachycardia, or ventricular fibrillation—occurred in 32.9% of patients. Myocardial work index (MWI) dispersion, expressed as standard deviation (SD) or Range, showed the highest single-parameter discrimination (AUC 0.848 and 0.859). Models combining MWI dispersion with right atrial (RA) area performed best (AUC 0.912 and 0.911). In multivariable logistic regression, both MWI dispersion and RA area were independently associated with VAs; odds ratios per 10-unit increase were 1.116 for MWI dispersion (SD), 1.030 for MWI dispersion (Range) and 1.276 per 1 cm^2^ of RA area. **Conclusions:** In this cohort, MWI dispersion showed the highest single-parameter discrimination for VAs among the echocardiographic variables tested, and its combination with RA area gave the best model. The two variables were independently associated with VAs in the same model. These exploratory findings are hypothesis-generating and require external validation before clinical application.

## 1. Introduction

Current evidence indicates that patients with heart failure with reduced ejection fraction (HFrEF) remain at substantial risk of ventricular arrhythmias (VAs) despite implantation of cardioverter defibrillator/cardiac resynchronisation therapy (ICD/CRT). In non-ischemic cardiac resynchronisation therapy–defibrillator (CRT-D) populations, the long-term incidence of VAs, defined as a sudden arrhythmic death, sustained VA or device-treated VA, is approximately 2–3% per year and is strongly modulated by response to resynchronization with CRT responders, exhibiting a markedly lower arrhythmic risk compared with non-responders [1]. Several structural and clinical variables have been proposed to elevate VAs risk among CRT recipients, including male sex, non-left bundle branch block (LBBB) morphology, enlarged left atrium (LA), persistent atrial fibrillation (AF), ischemic aetiology, and severely reduced left ventricular ejection fraction (LVEF) [2]. Myocardial work (MW) is a non-invasive echocardiographic parameter that combines left ventricular (LV) strain with estimated pressure to assess myocardial performance. Compared with global longitudinal strain (GLS), MW is less load-dependent and better reflects true myocardial function under varying afterload conditions. The global work index (GWI) represents the total work performed by the LV and is calculated as the area within the global pressure–strain loop [3,4]. The same calculation can be applied at the segmental level, yielding the segmental myocardial work index (MWI) for each of the 17 LV segments. The dispersion of segmental MWI values across the LV reflects the regional heterogeneity of myocardial work and indicates mechanical dyssynchrony.

Despite growing interest in non-invasive markers of mechanical dyssynchrony, MW dispersion has not yet been systematically evaluated as a potential predictor of VAs in patients with HFrEF. Current risk-stratification models rely heavily on LVEF [5], which has limited sensitivity for identifying individuals truly at risk. Because MW parameters provide a more detailed characterization of regional mechanical behaviour [3,6,7], we hypothesized that they may uncover arrhythmogenic substrates not captured by conventional echocardiographic measures. This gap in evidence inspired us to evaluate whether myocardial work dispersion measured before device implantation influences the occurrence of VAs in candidates for ICD/CRT. Understanding the relationship between mechanical dyssynchrony and arrhythmic risk may improve patient selection for device therapy and help refine risk-stratification strategies.

## 2. Materials and Methods

Patients with HFrEF who were admitted for an elective ICD/CRT implantation were prospectively enrolled in the study between 2021 and 2025. Exclusion criteria included a history of AF of any type, including paroxysmal AF, and lack of consent to participate. All patients underwent detailed clinical assessment and physical examination, as well as 12-lead electrocardiography (ECG) and transthoracic echocardiography (TTE). Patients with a history of AF (persistent, permanent and paroxysmal) were excluded because reliable QRS-gated speckle-tracking analysis of LA deformation and of segmental MW requires a stable sinus rhythm. Beat-to-beat cycle-length variability in AF would confound both atrial strain and pressure–strain loop measurements. Patients with paroxysmal AF were also excluded because episodes of AF during follow-up would additionally interfere with the detection and adjudication of ventricular arrhythmias by the device.

Data on medical therapy at the time of device implantation were collected from hospital records for every patient. We recorded beta-blockers, angiotensin-converting enzyme inhibitors, angiotensin receptor blockers, angiotensin receptor-neprilysin inhibitors, mineralocorticoid receptor antagonists, sodium-glucose co-transporter 2 inhibitors, loop diuretics, digoxin, amiodarone, class I antiarrhythmic drugs and statins.

### 2.1. Echocardiographic Assessment

All echocardiographic studies were carried out using a commercially available ultrasound platform equipped with a phased-array transducer (Vivid E95, GE Healthcare, Horten, Norway). Comprehensive two-dimensional imaging, Doppler measurements, and speckle-tracking analysis were performed in accordance with the latest recommendations from the American Society of Echocardiography (ASE) and the European Association of Cardiovascular Imaging (EACVI) [8,9,10].

Left ventricular linear parameters were assessed using the standard parasternal view. LV volumes were measured with the biplane Simpson method from apical 4- and 2-chamber views, and LVEF was calculated accordingly. Diastolic function was evaluated by transmitral Doppler flow (E and A waves, E/A ratio) and tissue Doppler–derived mitral annular velocities (e′). The E/e′ ratio was used as a non-invasive estimate of LV filling pressures in accordance with current ASE/EACVI recommendations [11].

Left atrial (LA) volume was measured in apical 4- and 2-chamber views and indexed to body surface area (left atrial volume index, LAVI). LA deformation was assessed by two-dimensional speckle tracking echocardiography (2D-STE) from apical views. The LA endocardial border was manually traced, with automated region-of-interest generation. Analyses were QRS-gated. Reservoir (LASr), conduit (LAScd), and contraction (LASct) strain components were derived in accordance with EACVI/ASE recommendations [12].

Left ventricular longitudinal strain was evaluated with 2D-STE in all apical chamber views, and GLS was calculated as the mean peak systolic strain across all 17 LV segments. Myocardial work parameters were estimated using non-invasive pressure–strain loop analysis, with brachial systolic pressure serving as a surrogate for peak LV pressure. Derived indices included global work index (GWI), global constructive work (GCW), global wasted work (GWW), and global work efficiency (GWE), following the methodology originally proposed by Russell et al. and later validated in several large-scale studies [3,13]. All post-processing was conducted offline using EchoPAC software (version 204, GE Healthcare, Horten, Norway).

Myocardial work index dispersion (MWI dispersion) was calculated from the 17 segmental MWI values obtained from the bullseye pressure–strain loop analysis, using two complementary approaches. MWI dispersion (standard deviation, SD) was calculated as the standard deviation of segmental MWI values across all 17 LV segments. MWI dispersion (Range) was defined as the difference between the maximum and minimum segmental MWI values. SD reflects the overall spread of segmental values around the mean, whereas Range captures the extreme inter-segment difference and is straightforward to interpret. Since these are novel indices and it remains unclear which metric better represents true MW dispersion, both complementary measures were reported. Analogous dispersion measurements (SD and Range) were calculated from segmental myocardial work efficiency (MWE) values. For comparison, peak strain dispersion (PSD) was assessed as a timing-based index of LV mechanical heterogeneity, corresponding to the parameter commonly referred to as mechanical dispersion (MD) in the arrhythmia literature, and was calculated as the standard deviation of the time from QRS onset to peak longitudinal strain across the 17 LV segments. PSD is therefore conceptually distinct from MWI dispersion, which quantifies the heterogeneity of segmental myocardial work (the pressure–strain loop area) rather than of contraction timing. Throughout, the timing-based parameter is termed PSD and the work-based parameters MWI dispersion (SD) and (Range). All myocardial work and dispersion measurements require an adequate acoustic window, because they are derived from speckle-tracking of all 17 LV segments.

### 2.2. Electrocardiographic Assessment

A standard 12-lead ECG was recorded electronically in all patients, and the recordings were subsequently analysed offline for heart rate (HR), PQ interval, P-wave duration, and QRS duration.

### 2.3. Follow-Up Assessment

Follow-up evaluations were performed at 1, 6, and 12 months during routine checks of the implanted device. Ventricular arrhythmias were defined as sustained ventricular tachycardia (sVT), non-sustained ventricular tachycardia (nsVT), or ventricular fibrillation (VF), diagnosed according to guidelines [14]. These data were established after analysis of intracardiac electrograms (IEGMs). Ventricular arrhythmias were diagnosed if the ventricular rate was above programmed VT and VF detection zones (ICD therapy zones programmed at physician discretion), after excluding episodes of supraventricular arrhythmias with high ventricular rate, artifacts or myopotentials. The composite arrhythmic endpoint (sVT, nsVT or VF) was pre-specified. Non-sustained VT was included deliberately. In patients with HFrEF, nsVT recorded by an implanted device is not an incidental finding but a documented spontaneous VA arising from the same electroanatomical substrate as sustained events, and it is recognised in current guidelines as a marker of arrhythmic risk and an indication for further evaluation [14]. Restricting the endpoint to device-treated events would additionally make it dependent on programmed detection zones and therapy settings, which were left to physician discretion, and would therefore measure device programming as much as arrhythmic burden. The endpoint used here reflects the occurrence of spontaneous VA rather than the decision to treat it. A transthoracic echocardiogram was also performed at the 12-month device check at this centre, but the follow-up echocardiographic data were not used in the present analysis. Three patients received a CRT-pacing (CRT-P) device. These devices store intracardiac electrograms and detect ventricular high-rate episodes in the same way as ICD and CRT-D devices, so the detection and adjudication of nsVT, sVT and VF were comparable. The only difference is that a CRT-P device cannot deliver antitachycardia pacing or a shock. Because our endpoint was the occurrence of a spontaneous arrhythmia and not the delivery of therapy, monitoring in these three patients was comparable with the rest of the cohort. The numbers of patients with nsVT, sVT and VF and with device therapies are reported separately in the Results.

### 2.4. Ethical Statement

All procedures were performed in compliance with the principles of Good Clinical Practice and the Declaration of Helsinki. The study protocol received approval from the ethics committee of the Military Institute of Medicine—National Research Institute (Resolution No. 23/WIM/2021, date of approval 16 June 2021). Written informed consent was obtained from every participant prior to enrolment.

### 2.5. Statistical Methods

All analyses were performed using Statistica 13.0 (TIBCO Software Inc., San Ramon, CA, USA). Distribution of continuous variables was assessed with the Shapiro–Wilk test. Between-group comparisons were conducted using the Mann–Whitney U test or Student’s *t*-test as appropriate, and categorical variables with the chi-square or Fisher’s exact test. Correlations were evaluated with Spearman’s rank correlation coefficient.

Univariable logistic regression was used to identify candidate predictors of ventricular arrhythmias. Variables that were statistically significant in univariable analysis and showed high discriminatory capacity (AUC) were considered for multivariable models. Given 28 events, the number of predictors was restricted to a maximum of 3–4 to avoid overfitting, and only non-collinear variables (low pairwise Spearman coefficients) were included. Multivariable models were constructed using a stepwise approach, and the best-performing models were selected based on R^2^, the Akaike Information Criterion (AIC), and AUC.

The outcome was analysed as the occurrence of a ventricular arrhythmia during a fixed follow-up period of one year. Logistic regression was therefore used instead of a time-to-event model. All patients had a complete 12-month follow-up, so there was no censoring before the end of follow-up, and the exact date of the first arrhythmic episode was not recorded for every patient. A Cox model was consequently not feasible, and the results should be read as the risk of having an arrhythmic event within one year rather than as the rate of events over time.

Receiver operating characteristic (ROC) curves were used to assess discriminatory performance, with optimal cut-offs identified by the Youden index. AUC values are reported with 95% confidence intervals. A two-sided *p*-value < 0.05 was considered statistically significant.

Model calibration was assessed by the Brier score, the Hosmer–Lemeshow goodness-of-fit test, and comparison of observed and predicted numbers of events across quintiles of predicted risk. To test whether the association between MWI dispersion and VAs depended on device type, an interaction term (MWI dispersion × CRT vs ICD) was added to the multivariable models. The best model was additionally re-fitted, with each of the following covariates added in turn: ischaemic aetiology, chronic kidney disease, LVEF, age, NT-proBNP and QRS duration. Strain and myocardial work values are reported as absolute values throughout.

Internal validation was performed by bootstrap resampling. First, the two final models were refitted in 1000 bootstrap samples, and the optimism in the area under the curve was estimated by the difference between the performance of each bootstrap model in its own sample and in the original data; the optimism-corrected AUC and the calibration slope are reported. Second, because the models were built from a larger set of candidate variables, the whole selection procedure was repeated inside the bootstrap. In 500 bootstrap samples, the best two-variable model was selected by AIC from twelve candidate predictors and evaluated in the original data, which gives an estimate of optimism that includes model selection. The frequency with which each variable was selected is also reported.

The incremental value of RA area was tested formally. The model with MWI dispersion alone was compared with the model containing MWI dispersion and RA area by the likelihood ratio test, and the difference in AUC between the two models was estimated with 2000 bootstrap resamples.

Three sensitivity analyses were pre-planned for the revision. The models were refitted with the endpoint restricted to sustained events (sVT or VF), after exclusion of the three CRT-P recipients, and with the type of implanted device (CRT versus ICD) added as a covariate.

Intra-observer reproducibility was assessed in 20 randomly selected patients, in whom the same investigator reanalysed the myocardial work data after several days, blinded to the original results. The reproducibility of global and segmental myocardial work indices was quantified using the intraclass correlation coefficient (ICC), Bland–Altman analysis, and the coefficient of variation.

## 3. Results

A total of 96 patients qualified for ICD/CRT were initially screened. After excluding four patients with follow-up below 1 year, three with poor echocardiographic image quality, and four without available follow-up data, 85 patients were included in the final analysis. Of these 85 patients, 53 (62.4%) received a cardiac resynchronisation device (50 CRT-D and 3 CRT-P) and 32 (37.6%) an ICD, in accordance with current guidelines [5].

Among 85 patients with HFrEF and implanted ICD/CRT devices, VAs, defined as sVT/nsVT/VF, were detected in 28 cases (32.9%) after 1 year of follow-up. The composite endpoint comprised nsVT in 24 patients, sVT in six and VF in three; these categories overlap, and nine patients had a sustained event (sVT or VF). Appropriate device therapies were delivered in 11 patients and an inappropriate therapy occurred in one patient. None of the three CRT-P recipients had an arrhythmic event.

No difference in the occurrence of VAs was observed between ischemic and non-ischemic aetiologies of HFrEF. Arrhythmic events were more frequent in men. However, sex was not a significant factor in the univariable regression, and its inclusion in the multivariable models did not improve their predictive performance.

Table 1 and Table 2 summarise the baseline characteristics of patients with and without VAs. The groups did not differ significantly in age, body mass index, or LV dimensions and volumes. In contrast, patients with VAs showed markedly altered atrial mechanics, with lower LASr and LASct values, accompanied by higher LA stiffness. Myocardial work indices also demonstrated greater heterogeneity in the arrhythmia group, reflected by increased dispersion metrics.

Medical therapy is shown in Table 1 and was available for 83 of the 85 patients. Almost all patients received guideline-directed medical therapy, and the two groups were well matched. A beta-blocker was given to 27 of 27 patients with VAs and 55 of 56 patients without VAs (*p* = 1.00), a mineralocorticoid receptor antagonist to 96.3% and 91.1% (*p* = 0.66), a sodium-glucose co-transporter 2 inhibitor to 74.1% and 83.9% (*p* = 0.37), and an inhibitor of the renin-angiotensin system to 100% and 94.6% (*p* = 0.55). Amiodarone was used in 7.4% and 10.7% (*p* = 1.00), and no patient received a class I antiarrhythmic drug. The only difference was more frequent use of a loop diuretic in patients with VAs (96.3% versus 71.4%, *p* = 0.01).

### 3.1. Predictive Performance of Myocardial Work Dispersion Parameters

Several variables reached statistical significance in the univariable regression analysis. Detailed results are presented in Table 3.

Among all single predictors, indices reflecting the heterogeneity of LV myocardial work demonstrated the highest discriminatory performance. The dispersion of myocardial work index (dispersion MWI (SD)) showed an AUC of 0.848 (95% CI: 0.768–0.927), and the dispersion MWI (Range) demonstrated an AUC of 0.859 (0.783–0.935), both markedly outperforming conventional echocardiographic markers. In contrast, standard parameters of LV size or diastolic function (e.g., LVDd, E/e′, E/A) exhibited poor to moderate predictive ability (AUC between 0.56 and 0.65).

Markers of atrial size and function showed intermediate performance, with RA area (AUC 0.784) and LA area (AUC 0.713) providing the best discrimination among non-LV myocardial work variables. Detailed data on AUC for all single parameters are presented in Table S1.

Dispersion MWI (SD) achieved a sensitivity of 74% and a specificity of 89% at a cut-off value of 408.9, while dispersion MWI (Range) showed similarly high accuracy, with a sensitivity of 72% and a specificity of 93% at a cut-off value of 1546 (Figure 1). In comparison, RA area exhibited more modest discrimination, with a sensitivity of 64% and a specificity of 81% (cut-off value 17.3).

### 3.2. Multivariable Models

The combination of MWI dispersion (SD or Range) and RA area was the most effective model, with the highest discrimination (AUC 0.912 and 0.911, respectively), the greatest explained variance (R^2^ 0.583 and 0.592), and the lowest AIC values.

Several additional variables (LAVI, V(TR), LA area, E/e′, LASr, LASct) modestly improved the discrimination of MW dispersion alone, typically achieving AUC values between 0.87 and 0.89; however, none outperformed the combined MWI dispersion + RA area model.

Across all alternatives, MWI dispersion assessed in both ways (MWI SD and MWI Range) remained in the best-performing models.

Table S2 presents the detailed parameters used to compare the predictive models.

Table 4 presents the detailed results of regression logistic analysis for both of the best models. Because the unit change of both dispersion parameters is very small, the per-1-unit odds ratios in Table 4 appear modest despite a strong clinical effect; when rescaled to a 10-unit increase, MWI dispersion (SD) and (Range) were associated with an 11.6% and 3.0% higher odds of VAs, respectively. The odds ratios for MWI dispersion and for RA area are expressed in different units (work index units and cm^2^) and cannot be compared directly with each other. When the two variables are compared on the same scale, that is, by their discrimination as single predictors, MWI dispersion performed better than RA area (AUC 0.848 and 0.859 versus 0.784). Both variables remained independently associated with VAs after mutual adjustment, in both parameterizations.

Intra-observer reproducibility was good to excellent for all myocardial work indices (ICC 0.931–0.991 for global and 0.967–0.979 for segmental measures; coefficients of variation 2.0–9.6%), with only minimal mean differences on Bland–Altman analysis.

Calibration of the two best models was good. The Brier score was 0.111 for both the model containing MWI dispersion (SD) and the model containing MWI dispersion (Range), and the Hosmer–Lemeshow test showed no evidence of miscalibration (χ^2^ 0.39, df 3, *p* = 0.94 and χ^2^ 0.47, df 3, *p* = 0.93, respectively). Observed and predicted numbers of events agreed closely across quintiles of predicted risk; in the highest quintile, 15 events were observed versus 14.4 and 14.5 predicted, and in the lowest two quintiles 1 event was observed versus 1.1 and 1.0 predicted.

Internal validation and sensitivity analyses: In 1000 bootstrap samples, the apparent AUC of 0.912 for the model with MWI dispersion (SD) and RA area fell to an optimism-corrected AUC of 0.908, with a calibration slope of 0.949. For the model with MWI dispersion (Range), the apparent AUC of 0.911 fell to 0.906, with a calibration slope of 0.931.

When the whole selection procedure was repeated inside the bootstrap, using twelve candidate predictors and 500 resamples, the apparent AUC of the selected models was 0.906 and the optimism-corrected AUC was 0.881, with a calibration slope of 0.811. A myocardial work dispersion variable was selected in 100% of the bootstrap samples, MWI dispersion (SD or Range) in 99% and RA area in 56%. These figures show that the optimism generated by model selection is real but modest and that MWI dispersion is a stable component of the model, whereas the second variable is less stable.

The incremental value of RA area was confirmed formally. Adding RA area to MWI dispersion (SD) improved the fit significantly (likelihood ratio χ^2^ 12.67, *p* = 0.0004) and increased the AUC from 0.848 to 0.912; the bootstrap difference in AUC was 0.068 (95% CI 0.021–0.133). For MWI dispersion (Range) the corresponding values were χ^2^ 11.86, *p* = 0.0006, AUC 0.859 to 0.911, and a difference in AUC of 0.056 (95% CI 0.014–0.113).

The proportion of resynchronisation devices differed between the groups. When the type of implanted device was added to the models as a covariate, the results did not change: the AUC was 0.913 for MWI dispersion (SD) and 0.914 for MWI dispersion (Range), device type was not significant (*p* = 0.98 and *p* = 0.83), and both MWI dispersion (*p* = 0.0004 and *p* = 0.0003) and RA area (*p* = 0.002 and *p* = 0.003) remained significant. Excluding the three CRT-P recipients did not change the results either (*n* = 82, AUC 0.915 and 0.918, both variables *p* < 0.01).

When the endpoint was restricted to sustained events only, there were nine such events. The models retained moderate to good discrimination (AUC 0.798 for MWI dispersion (SD) and 0.816 for MWI dispersion (Range)), and MWI dispersion remained significant (*p* = 0.03 for both), but RA area did not (*p* = 0.28 and *p* = 0.30). With only nine events, this analysis is clearly underpowered and is presented as exploratory.

### 3.3. Device Type and Adjustment for Potential Confounders

The type of the implanted device did not influence the occurrence of VAs: the interaction term (MWI dispersion × CRT vs ICD) was not significant either for MWI dispersion (SD) (*p* = 0.23) or for MWI dispersion (Range) (*p* = 0.38). When the best model (MWI dispersion + RA area) was re-fitted with additional covariates, MWI dispersion remained an independent predictor after adjustment for ischaemic aetiology (AUC 0.914, *p* = 0.0003), chronic kidney disease (AUC 0.914, *p* = 0.0003), LVEF (AUC 0.915, *p* = 0.0003) and age (AUC 0.913, *p* = 0.0003), and also after adjustment for NT-proBNP in the subgroup with available measurements (*n* = 67, AUC 0.916, *p* = 0.002) and for QRS duration (*n* = 79, AUC 0.922, *p* = 0.004). Adjusting simultaneously for ischaemic aetiology, LVEF and chronic kidney disease did not change the result (AUC 0.916, *p* = 0.0003). Response to CRT was not part of the present analysis, which was based only on the echocardiographic study performed before implantation.

## 4. Discussion

In this prospective cohort of HFrEF patients with ICD/CRT, dispersion of myocardial work was associated with the occurrence of VAs and showed the highest discrimination of all echocardiographic variables tested. Both metrics, MWI standard deviation and range, performed consistently, which suggests that MW dispersion may be a useful marker of arrhythmic risk. Because the study is small, single-centre and exploratory, this association should be regarded as hypothesis-generating and needs confirmation in independent cohorts. Right atrial (RA) area yielded a numerically larger odds ratio per unit increase, but this reflects the difference in measurement scale between the two variables—RA area is expressed in cm^2^ over a narrow range, whereas MWI dispersion spans several hundred units—rather than a stronger underlying association; on the scale-independent AUC metric, RA area (0.784) discriminated less well than MWI dispersion. Models combining MWI dispersion with RA area performed best, and RA area provided the largest additional gain in discrimination when combined with MWI dispersion (AUC 0.911–0.912), suggesting that the two parameters capture complementary, left ventricular mechanical and right-sided structural, components of arrhythmic risk rather than a single dominant substrate. Data on the role of the right atrium in predicting ventricular arrhythmias in the HFrEF population remain limited. Previous studies assessing right atrial size in this population have focused mainly on all-cause mortality rather than on ventricular arrhythmia risk as a separate endpoint [15]. The present study is therefore among the few in which right atrial area has been directly related to the risk of ventricular arrhythmias in patients with HFrEF, partially addressing this gap in the literature, although confirmation in larger studies is required.

The order of the study procedures deserves a comment. Myocardial work was measured one day before implantation, the device was implanted afterwards, and the arrhythmic events were recorded during the following year. The measurement therefore precedes both the device and the events, and the device itself cannot have influenced the value of MWI dispersion. What we describe is the relationship between myocardial work dispersion measured before implantation and ventricular arrhythmias occurring after implantation. This temporal order supports the direction of the association, but it does not prove causality, because the study is observational and the arrhythmogenic substrate was not characterised directly.

Combining MWI dispersion with RA area provided the highest discriminative accuracy while preserving model simplicity, highlighting the role of LV mechanical heterogeneity and right-sided remodelling in VAs in advanced systolic HF.

The large meta-analysis demonstrated that LV mechanical dispersion (MD) is strongly associated with VAs in various cardiac diseases and provides better risk stratification than LVEF and GLS [16]. Haugaa et al. [17] showed that increased MD characterises post-myocardial infarction patients at higher risk of VAs and that echocardiographic assessment of MD, together with global strain, may improve risk stratification beyond LV systolic dysfunction, particularly in patients with preserved LV function. Perry et al. [18] showed that LVEF alone is inadequate for predicting VAs and sudden cardiac death and that MD may improve risk stratification, particularly in patients not meeting LVEF-based criteria for defibrillator therapy. This classic, timing-based mechanical dispersion corresponds to the PSD parameter in the present study and should be distinguished from MWI dispersion, which reflects the heterogeneity of regional MW rather than of contraction timing. In our cohort, PSD discriminated arrhythmic patients less well than MWI dispersion (Table 2 and Table 3), which may seem unexpected given that work is derived from the strain curve combined with estimated pressure. A plausible explanation is that timing-based dispersion captures only the temporal sequence of peak deformation, whereas MWI dispersion additionally incorporates regional load through the pressure-strain integral, weighting each segment by the work it performs. It may therefore characterise the underlying arrhythmogenic substrate—regional scar, fibrosis, and inhomogeneous loading—more closely than the timing of peak strain alone.

Our study applies a pathophysiology-based approach to arrhythmic risk stratification by showing that MW dispersion is associated with VAs in patients with HFrEF. Reduced global MW has been associated with a higher risk of adverse cardiovascular outcomes [18,19,20]. A plausible, though unconfirmed, explanation is that increased MWI dispersion reflects heterogeneous myocardial loading, regional fibrosis or scar, and impaired excitation–contraction coupling—substrates classically implicated in re-entrant VAs—though none of these tissue-level mechanisms were directly assessed in this study. The similar predictive performance of both SD- and Range-based dispersion metrics suggests that global mechanical heterogeneity, rather than isolated regional abnormalities, is central to arrhythmic risk in HFrEF. This interpretation is a possible explanation of our findings and was not tested directly in the present study, because no tissue characterisation or electroanatomical data were available.

A specific consideration is that most patients were candidates for CRT, in whom conduction delay and electrical dyssynchrony could, in principle, increase segmental work heterogeneity and inflate MWI dispersion, raising the question of whether its association with VAs merely reflects a more dyssynchronous substrate. Among the 85 analysed patients, a resynchronisation device was implanted in 13 of 28 patients with VAs (46.4%) and in 40 of 57 patients without VAs (70.2%); this difference did not reach statistical significance (*p* = 0.06). More importantly, the association between MWI dispersion and VAs did not differ by device type: the interaction term (MWI dispersion × CRT vs ICD) was not significant (*p* = 0.23 for SD and *p* = 0.38 for Range). This argues against the predictive value of MWI dispersion being explained by electrical dyssynchrony alone and is compatible with the view that it also reflects an arrhythmogenic substrate, regional fibrosis or scar, heterogeneous loading, and impaired excitation–contraction coupling, beyond simple conduction delay, although these components cannot be separated in the present data. Finally, although CRT response modulates subsequent arrhythmic risk, with responders showing lower VA rates than non-responders [1], our baseline assessment was not designed to test this. A follow-up echocardiogram is performed at the 12-month device check in our centre, but response to CRT was not part of the present analysis, which was based only on the study performed before implantation. These data are still being collected and analysed and will be the subject of a separate analysis. Whether baseline MWI dispersion predicts CRT response warrants dedicated study.

Contemporary data show that many patients receiving ICDs never experience appropriate therapies, while others with only moderately reduced LVEF remain at high arrhythmic risk [21,22]. In our previous study, LVEF was not associated with the risk of appropriate ICD interventions but remained an independent predictor of long-term all-cause mortality [23]. In our cohort, MWI dispersion indicated that arrhythmogenesis reflects mechanical and electrical instability rather than global systolic dysfunction alone.

MW indices have also been linked to mortality, hospitalization, and adverse remodelling in HF [18,19,24], supporting their role as an imaging biomarker for refining ICD eligibility.

Medical therapy is unlikely to explain the difference in arrhythmic events between the groups. Guideline-directed treatment was used in almost all patients, and the proportions of beta-blockers, renin-angiotensin system inhibitors, mineralocorticoid receptor antagonists, sodium-glucose co-transporter 2 inhibitors and amiodarone were similar in both groups. Loop diuretics were used more often in patients with VAs, which probably reflects more advanced congestion rather than a proarrhythmic effect and is consistent with the larger RA area in this group.

A clinically relevant finding is the independent association of RA area with VAs in this model and its synergistic interaction with MWI dispersion. Right atrial enlargement reflects chronically elevated right-sided filling pressures and advanced HF severity [25], and right-sided remodelling marks disease progression and adverse outcomes in HFrEF even without atrial arrhythmias [26]. RA area should therefore not be read as a direct arrhythmic marker. It is more likely a marker of overall heart failure severity, right-sided congestion and right ventricular dysfunction, and this is the most plausible reason why it adds information to a measure of LV mechanical heterogeneity. It may seem more natural that LA size or a marker of diastolic dysfunction would carry this information, and LA area, LAVI and E/e′ were indeed associated with VAs in the univariable analysis. In the multivariable models, however, RA area performed better. A likely explanation is that LA size and the diastolic indices describe the consequences of left-sided filling pressures, which are already partly captured by the left ventricular variable in the model, whereas RA area reflects the further, right-sided stage of the disease and therefore adds information that the left-sided variables do not. RA area is also a simple and robust measurement, which may make it more reproducible than E/e′ or LA strain in this population.

The combination of LV mechanical heterogeneity and RA enlargement likely captures two complementary arrhythmogenic pathways: intrinsic myocardial instability and advanced haemodynamic remodelling.

Our results should be read as exploratory. The cohort is small, with 85 patients and 28 events, and the models were built from a larger set of echocardiographic variables in the same data, which generates optimism. We therefore validated the models internally by bootstrapping. When the models were refitted in bootstrap samples, the optimism-corrected AUC was 0.908 and 0.906, close to the apparent values. When the entire selection procedure was repeated inside the bootstrap, the optimism-corrected AUC fell from 0.906 to 0.881 and the calibration slope was 0.811, which indicates that part of the apparent performance does come from model selection and that the coefficients would need shrinkage in a new cohort. MWI dispersion was nevertheless selected in 99% of the bootstrap samples, whereas RA area was selected in 56%, so the dispersion component of the model is stable and the second component is not. In addition, the sex distribution was very imbalanced, with only one woman among the patients with VAs, so no conclusion can be drawn for women. For these reasons, we present the models as hypothesis-generating and not as a validated risk tool.

The absence of cardiac magnetic resonance is a further reason for caution. Myocardial scar and fibrosis could plausibly cause both increased regional heterogeneity of work and increased arrhythmic risk, so MWI dispersion may be acting partly as a mechanical surrogate of scar. Whether it carries independent information beyond late gadolinium enhancement cannot be answered with our data. Until this is tested and until the model is validated in an independent cohort, we do not propose that MWI dispersion should be added to routine pre-implantation assessment.

Combined with routine assessment of RA size, MWI dispersion may help identify HFrEF patients at increased arrhythmic risk despite similar LVEF, refine individualized risk stratification, and support shared decision-making on device therapy and rhythm surveillance. The character of our results is, however, exploratory and hypothesis-generating, and they require confirmation in independent cohorts. Before MWI dispersion can be considered for routine pre-implantation assessment, the model has to be validated externally.

Apart from that, several limitations should be acknowledged. This was a single-centre study with a relatively small sample size, which may limit external validity. In addition, sex distribution was markedly imbalanced, with only one female patient in the VA group, which limits any sex-specific inference and means a potential role of sex in arrhythmic risk cannot be excluded in this cohort. As proper interpretation of MW indices requires stable sinus rhythm, we intentionally excluded patients with AF of any type, including paroxysmal AF, which restricts the applicability of our findings to this common HF subgroup; whether MWI dispersion has any value in patients with AF is unknown. A similar limitation must be acknowledged in patients with a suboptimal acoustic window: myocardial work analysis requires adequate imaging of all 17 LV segments (three patients were excluded for poor image quality), and myocardial work assessment also depends on non-invasive blood pressure estimation, which may introduce measurement variability. This suggests that in real-world practice, a considerable proportion of HFrEF patients, particularly those with obesity, chronic obstructive pulmonary disease, or prior thoracic surgery, may not be eligible for this form of risk stratification, which could limit the generalizability and clinical applicability of our findings. Ventricular arrhythmic events were defined based on device-detected arrhythmias rather than arrhythmic mortality. The timing of arrhythmic episodes was not recorded for every patient, so a time-to-event analysis could not be performed, and the outcome was analysed as the occurrence of an event within the predefined one-year follow-up. Information on changes in medical treatment after an arrhythmic event was not collected systematically and could not be analysed. The composite endpoint combined nsVT, sVT and VF, and these events differ in clinical importance. In a sensitivity analysis restricted to sustained events, MWI dispersion remained significant, but only nine such events occurred and RA area was no longer significant, so that analysis is underpowered and should be read with caution. Detection zones were programmed at the discretion of the treating physician, which is a further reason why the endpoint was defined as the occurrence of a spontaneous arrhythmia rather than as a device-treated arrhythmia. An additional limitation of the study is the lack of inter-observer reproducibility assessment. Consequently, despite the good intra-observer agreement observed, the consistency of MW measurements and derived dispersion indices across different observers has not yet been established. This is a relevant limitation for a new imaging-derived index, and inter-observer reproducibility should be reported before MWI dispersion is considered for practice. The impact of CRT response on the subsequent occurrence of VAs could not be assessed, and the dyssynchrony-related and substrate-related components of MWI dispersion could not be fully disentangled. A QRS-adjusted analysis in a larger cohort would help to separate these contributions. An important limitation is the absence of cardiac magnetic resonance imaging. Late gadolinium enhancement–defined myocardial scar is the strongest established imaging predictor of ventricular arrhythmias in this population, and increased MWI dispersion may in part act as a mechanical surrogate of regional scar rather than as an independent substrate marker. Scar is not the only possible cause. Increased dispersion of segmental work may also result from myocardial ischaemia, which changes regional contraction and regional loading without permanent tissue loss, and our study included a majority of patients with ischaemic heart disease. Ischaemia and scar cannot be separated with echocardiography alone. Whether MWI dispersion retains predictive value after adjustment for scar burden cannot be determined from the present data and requires a study with combined echocardiographic and CMR phenotyping. Finally, the models were developed and evaluated within the same small cohort using a data-driven selection strategy, so the reported discrimination should be regarded as exploratory and requires external validation before clinical use. Accordingly, external validation of the proposed model in a larger, independent cohort is planned, and the present results should be regarded as hypothesis-generating until that validation is completed.

## 5. Conclusions

In this prospective single-centre cohort, myocardial work index dispersion showed the highest single-parameter discrimination for ventricular arrhythmias defined as sVT/nsVT/VF, regardless of the metric used (SD or Range), and right atrial area provided the largest incremental improvement when added to it. Both variables were independently associated with VAs in the same model. These observations suggest that left ventricular mechanical heterogeneity and right-sided atrial remodelling capture complementary components of arrhythmic susceptibility. Given the limited sample size and the exploratory nature of the model-building process, these findings should be confirmed in larger, externally validated cohorts before being applied to clinical decision-making.

## Figures and Tables

**Figure 1:**
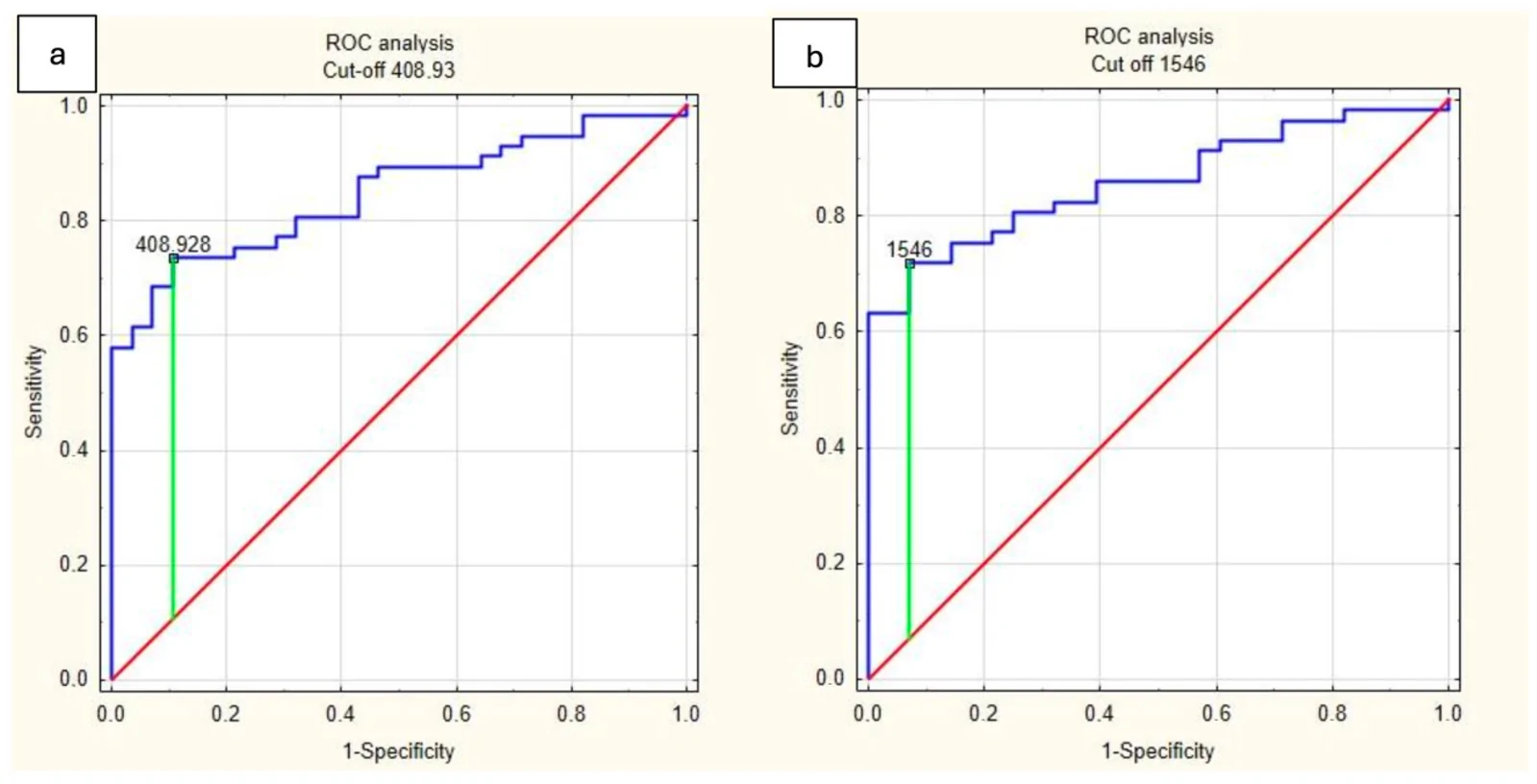
Receiver operating characteristic (ROC) curves illustrating the discriminatory performance of dispersion parameters of the myocardial work index (MWI): (**a**) dispersion MWI (SD) and (**b**) dispersion MWI (Range).

**Table 1:** Clinical and ECG characteristics of both study groups.

Variable	Patients with VAs		Patients Without VAs		*p*
	*n*		*n*		
Gender female, *n* (%)	28	1 (3.57)	57	13 (22.8)	0.03
Age, years	28	63 (55.5–73.5)	57	66 (58–74)	0.57
BMI, kg/m^2^	28	26.5 (24.5–29.4)	57	26.4 (24.5–29)	0.89
Ischemic HF	28	17 (60.7)	57	38 (66.7)	0.63
Hypertension	28	17 (60.7)	57	40 (70.2)	0.46
Diabetes mellitus	28	10 (35.7)	57	22 (38.6)	0.98
Stroke	28	4 (14.3)	57	5 (8.8)	0.47
Myocardial infarction	28	19 (67.9)	57	37 (64.9)	0.98
CABG	28	5 (17.9)	57	6 (10.5)	0.49
PCI	28	19 (67.9)	57	32 (56.1)	0.35
CAD	28	22 (78.6)	57	41 (71.9)	0.60
Vascular disease	28	24 (85.7)	57	43 (75.4)	0.39
Dyslipidaemia	28	20 (71.4)	57	41 (75.9)	0.79
CKD	28	9 (32.1)	57	12 (21.1)	0.29
Smoking	28	15 (53.6)	57	24 (43.6)	0.49
LBBB	28	11 (39.3)	57	22 (37.9)	0.91
CRT (CRT-D or CRT-P)	28	13 (46.4)	57	40 (70.2)	0.06
NT-proBNP, pg/mL	20	1976 (701–7232)	47	1397 (748.5–2928)	0.17
Medical therapy at implantation, *n* (%)					
Beta-blocker	27	27 (100)	56	55 (98.2)	1.00
RAS inhibitor (ACEI/ARB/ARNI)	27	27 (100)	56	53 (94.6)	0.55
MRA	27	26 (96.3)	56	51 (91.1)	0.66
SGLT2 inhibitor	27	20 (74.1)	56	47 (83.9)	0.37
Loop diuretic	27	26 (96.3)	56	40 (71.4)	0.01
Amiodarone	27	2 (7.4)	56	6 (10.7)	1.00
Class I antiarrhythmic drug	27	0 (0)	56	0 (0)	1.00
Digoxin	27	1 (3.7)	56	0 (0)	0.33
Statin	27	25 (92.6)	56	50 (89.3)	1.00
ECG parameters, median (IQR)					
HR, beats/min	25	66 (62–73)	53	69 (60–80)	0.31
P wave duration, ms	24	120 (101–135)	51	112 (100–125)	0.14
PQ interval, ms	25	188 (170–196)	52	174 (162–193)	0.10
QRS duration, ms	26	148 (112–162)	53	132 (107–160)	0.57

Abbreviations: ACEI—angiotensin converting enzyme inhibitor; ARB—angiotensin receptor blocker; ARNI—angiotensin receptor-neprilysin inhibitor; BMI, body mass index; CABG, coronary artery by-pass; CAD, coronary artery disease; CKD, chronic kidney disease; CRT, cardiac resynchronisation therapy; HF, heart failure; HR, heart rate; LBBB, left bundle branch block; MRA, mineralocorticoid receptor antagonist; NT-proBNP, N-terminal pro-B-type natriuretic peptide; PCI, percutaneous coronary intervention; RAS, renin-angiotensin system; SGLT2, sodium-glucose co-transporter 2, VAs—ventricular arrythmias.

**Table 2:** Echocardiographic characteristics of the two study groups.

Variable	Patients with VAs		Patients Without VAs		*p*
	*n*	Median (IQR)	*n*	Median (IQR)	
RVDd, mm	28	33.5 (30–37.7)	57	31 (29–33)	0.004
LVDd, mm	28	64.9 (60–72)	57	63 (60–68)	0.31
LVSd, mm	28	56 (52.5–63)	57	54 (49.9–60)	0.20
LVMI, g/m^2^	26	150.3 (127.7–174.2)	53	139.7 (131–173.8)	0.89
LASr, %	28	12 (6.5–16.5)	57	17 (14–21)	0.0002
LASct, %	28	7.5 (2.5–12)	57	13 (9–16)	0.0001
LAScd, %	28	4 (3–5.5)	57	4 (3–7)	0.89
LASI	27	1.2 (0.7–3.7)	57	0.72 (0.46–1.13)	0.003
LAEF, %	28	35 (21.5–44.5)	57	44 (35–50)	0.002
LVEDVi, mL/m^2^	28	102.9 (75.5–126.7)	57	92.2 (76.6–106.4)	0.26
LVESVi, mL/m^2^	28	72.6 (52.3–84.9)	57	61.7 (52.1–76.4)	0.23
SV, mL	28	56 (46.5–79.5)	57	54 (45–61)	0.28
GCW, mmHg%	28	856.5 (696–1094)	57	1099 (920–1354)	0.001
GWW, mmHg%	28	246 (154.5–372.5)	57	360 (263–496)	0.001
GWI, mmHg%	28	629.5 (452.5–806.5)	57	746 (584–962)	0.04
GWE, %	28	78.5 (71.5–85)	57	76 (67–81)	0.15
Dispersion MWI (SD)	28	523.5 (372.3–657.4)	57	310.5 (264.2–377.3)	<0.0001
Dispersion MWI (Range)	28	1912.5 (1402–2555)	57	1104.5 (866.5–1362)	<0.0001
Dispersion MWE (SD)	28	19.3 (14.6–24.9)	57	16.3 (12.9–20.8)	0.04
Dispersion MWE (Range)	28	66 (46–80)	57	59 (43–67)	0.05
PSD, ms	28	108.3 (76.1–156.1)	57	86.4 (69.8–126.9)	0.06
LA area, cm^2^	28	28.6 (24–34.1)	57	23.9 (21–26.9)	0.001
RA area, cm^2^	28	18.8 (15.8–22.8)	57	14.7 (13.2–16.9)	<0.0001
LAVI, mL/m^2^	28	53.3 (39.7–62.7)	57	42.5 (37–47.3)	0.03
LV GLS %	28	6.5 (5.3–8.8)	57	8 (6.6–9.8)	0.008
TAPSE, mm	28	18 (14–21)	57	20 (17–23)	0.06
S′ RV, cm/s	28	9 (7–10.5)	57	11 (9–12)	0.03
V (TR), m/s	28	2.8 (2.2–3.3)	50	2.3 (2–2.6)	0.003
e′ avg (cm/s)	27	4 (3–4.5)	57	4 (3.5–5)	0.30
E/e′ avg	27	14.8 (11.1–23.2)	57	11.4 (9.1–16.8)	0.04
E, cm/s	27	62 (39–91)	57	45 (41–63)	0.25
A, cm/s	27	57 (41–79)	57	73 (61–84)	0.04
E/A	27	0.98 (0.56–2.17)	57	0.63 (0.53–0.82)	0.04
LVEF, %	28	31 (24.5–35)	57	32 (27–36)	0.38

Abbreviations: E/A, ratio of early (E) to late (A) diastolic transmitral flow velocity; E/e′ avg, average E to e′ ratio; GCW, global constructive work; GWE, global work efficiency; GWI, global work index; GWW, global wasted work; IQR, interquartile range; LA, left atrium; LAEF, left atrial emptying fraction; LAScd, left atrial conduit strain; LASct, left atrial contraction strain; LASI, left atrial stiffness index (E/e′/LASr); LASr, left atrial reservoir strain; LAVI, left atrial volume index; LVDd, left ventricular end diastolic diameter; LVEDVi, left ventricular end diastolic volume index; LVEF, left ventricular ejection fraction; LVESVi, left ventricular end systolic volume index; LVMI, left ventricular mass index; LVSd, left ventricular end systolic diameter; LV GLS, left ventricle global longitudinal strain; MWE, myocardial work efficiency; MWI, myocardial work index; PSD, post systolic dispersion (timing-based mechanical dispersion); RA, right atrium; RVDd, right ventricular end diastolic diameter; SD, standard deviation; SV, stroke volume; S′ RV, right ventricular systolic velocity; TAPSE, tricuspid annular plane systolic excursion; V (TR), tricuspid regurgitation velocity.

**Table 3:** Univariable logistic regression analysis results.

Variable	OR	95% Cl	*p*
Gender (Female)	0.125	0.016–1.013	0.05
RVDd, mm	1.274	1.098–1.478	0.001
LASr, %	0.855	0.781–0.935	0.0006
LASct, %	0.834	0.755–0.921	0.0004
LASI (LA stiffness index, E/e′/LASr)	1.735	1.192–2.524	0.004
GCW, mmHg%	0.998	0.996–0.999	0.01
GWW, mmHg%	1.004	1.001–1.008	0.01
GWI, mmHg%	0.998	0.996–0.999	0.05
Dispersion MWI (SD)	1.011	1.005–1.016	0.0001
Dispersion MWI (Range)	1.003	1.002–1.004	<0.0001
Dispersion MWE (SD)	1.091	1.007–1.181	0.03
Dispersion MWE (Range)	1.026	0.999–1.053	0.05
LA area, cm^2^	1.131	1.044–1.225	0.002
RA area, cm^2^	1.333	1.146–1.550	0.0002
LAVI, mL/m^2^	1.038	1.006–1.072	0.02
GLS LV, %	0.858	0.727–1.012	0.07
S′ RV, cm/s	0.839	0.698–1.009	0.06
V(TR), m/s	3.683	1.543–8.789	0.003
E/e′ avg	1.070	1.003–1.142	0.04
A, cm/s	0.976	0.956–0.996	0.02
E/A	1.975	1.169–3.337	0.01
PSD, ms	1.012	1.001–1.023	0.05

Abbreviations: see Table 1 and Table 2.

**Table 4:** Detailed results of the multivariable logistic regression analysis for both of the best models.

Parameter	OR (95% CI)	*p*
Dispersion MWI (SD) and RA area		
Dispersion MWI (SD)	1.011 (1.005–1.017)	<0.0001
RA area	1.276 (1.083–1.503)	0.004
Dispersion MWI (Range) and RA area		
Dispersion MWI (Range)	1.003 (1.001–1.005)	<0.0001
RA area	1.295 (1.093–1.534)	0.003

Abbreviations: see Table 1 and Table 2.

## Data Availability

The data underlying this article are available from the corresponding author upon reasonable request. Due to the sensitive nature of the data, they are not publicly available.

## Supplementary Materials

The following supporting information can be downloaded at https://www.mdpi.com/article/10.3390/biomedicines14102159/s1, Table S1. AUC results for individual parameters. Table S2. Detailed parameters used to compare the predictive models.
