# Supplementary material for: Myocardial Work Index Dispersion as a Potential Predictor of Ventricular Arrhythmias in Patients with Heart Failure with Reduced Ejection Fraction
Source: Biomedicines. 2026 Sep 24;14(10):2159. doi: 10.3390/biomedicines14102159 (PMC13701602; doi:10.3390/biomedicines14102159)
Supplement: Supplementary file 1 [file biomedicines-14-02159-s001.zip › Table S1.pdf]

**Parameter**	**AUC**	**95% CI**
RVDd, mm	0.693	0.564-0.822
RA area, cm^2	0.784	0.679-0.888
LVDd, mm	0.568	0.431-0.705
LASr, %	0.748	0.639-0.857
LASct, %	0.759	0.653-0.865
GCW, mmHg%	0.711	0.589-0.832
GWW, mmHg%	0.714	0.592-0.837
GWI, mmHg%	0.638	0.511-0.765
GWE, %	0.596	0.467-0.726
Dispersion MWI (SD)	0.848	0.768-0.927
Dispersion MWI (Range)	0.859	0.783-0.935
Dispersion MWE (SD)	0.636	0.515-0.757
Dispersion MWE (Range)	0.633	0.509-0.756
PSD, ms	0.626	0.506-0.746
LA area, cm^2	0.713	0.590-0.836
V (TR), m/s	0.7	0.575-0.824
E/e' avg	0.638	0.509-0.767
E/A	0.638	0.502-0.774
QRS duration, ms	0.647	0.517-0.777
Abbreviations: see Table 1 and 2.		
