# Supplementary material for: Myocardial Work Index Dispersion as a Potential Predictor of Ventricular Arrhythmias in Patients with Heart Failure with Reduced Ejection Fraction
Source: Biomedicines. 2026 Sep 24;14(10):2159. doi: 10.3390/biomedicines14102159 (PMC13701602; doi:10.3390/biomedicines14102159)
Supplement: Supplementary file 1 [file biomedicines-14-02159-s001.zip › Table S2.pdf]

Model	Predictors	AIC	R^2	AUC
Dispersion MWI (SD)				0.848 (0.768-0.927)
+	RA area, LASr, V(TR)	65.484	0.575	0.907 (0.840-0.974)
	RA area	67.620	0.583	0.912 (0.849-0.975)
	LAVI	75.824	0.501	0.875 (0.802-0.948)
	RVDd	75.124	0.508	0.883 (0.809-0.956)
	E/e' avg	76.539	0.454	0.850 (0.771-0.929)
	E/A	76.540	0.455	0.850 (0.771-0.929)
	LA area	75.082	0.509	0.883 (0.811-0.954)
	V (TR)	68.432	0.544	0.883 (0.808-0.958)
	LASr	75.127	0.508	0.870 (0.797-0.944)
	LASct	74.741	0.512	0.870 (0.796-0.945)
	Gender, RA area	67.620	0.583	0.912 (0.849-0.975)
	Gender	78.289	0.453	0.848 (0.768-0.927)
Dispersion MWI (Range)				0.859 (0.783-0.935)
+	RA area	66.638	0.592	0.911 (0.849-0.973)
	LA area	73.646	0.523	0.893 (0.827-0.960)
	V (TR)	66.253	0.567	0.895 (0.826-0.964)
Abbreviations: AIC, Akaike Information Criterion; AUC, area under curve; see Table 1.				
